# An analysis on the entity annotations in biological corpora

**DOI:** 10.12688/f1000research.3216.1

**Published:** 2014-04-25

**Authors:** Mariana Neves

**Affiliations:** 1 Hasso-Plattner-Institut, Potsdam Universität, Potsdam, Germany

## Abstract

Collection of documents annotated with semantic entities and relationships are crucial resources to support development and evaluation of text mining solutions for the biomedical domain. Here I present an overview of 36 corpora and show an analysis on the semantic annotations they contain. Annotations for entity types were classified into six semantic groups and an overview on the semantic entities which can be found in each corpus is shown. Results show that while some semantic entities, such as genes, proteins and chemicals are consistently annotated in many collections, corpora available for diseases, variations and mutations are still few, in spite of their importance in the biological domain.

## Introduction

Annotated collections of documents are crucial components for developing new methods in text mining, such as extraction of named entities and relationships from the scientific literature. This lies in the fact that supervised learning systems need to rely on annotated documents to train the algorithms, and therefore, “learn” how to efficiently perform a certain task. Additionally, use of a standard collection of documents is practically the only way of performing an unbiased comparison between different systems for a particular task.

In natural language processing (NLP), a corpus can be defined as a collection of documents which usually belongs to a particular topic and that has been annotated according to a pre-defined schema. When annotating semantic information, a schema is usually composed of some entities (e.g., genes, proteins), and optionally, relationships (e.g., protein-protein interactions, gene-disease relationships). The number of documents may vary from a couple of full text documents ^1, 2^, to hundreds of abstracts ^3^ or thousands of sentences ^4^.

A schema can be composed of an arbitrary list of annotation types or based on terms pertaining to one or more ontologies. For example, the Variome ^1^ and the CellFinder ^2^ corpora contain annotations for a pre-defined list of entities, such as genes/proteins, cell lines, diseases and mutations. On the other hand, the CRAFT corpus ^5^ includes annotations according to concepts in seven ontologies and terminologies to allow a better identification of the annotations and their interoperability with other biomedical resources. The annotation schema is usually part of a comprehensive guideline document in which more details of the annotation process are described, such as an overview of the concepts, the provenance of the documents and examples of situations where the annotation should (or should not) be carried out.

Corpora are usually constructed for training or evaluation purposes during the development of a particular system (e.g., Gerner *et al.* ^6^) but are often also created in the context of a challenge or shared task (e.g., Krallinger *et al.* ^7^) to foster improvements on a particular task and allow comparison between different solutions. Corpora are usually manually annotated by human experts in a particular field or automatically derived using NLP techniques. When manually constructed by one or more annotators, it receives the denomination of a gold-standard corpus. In this process, annotators are required to carefully read the texts and manually annotate the text according to the pre-defined schema. This annotation process is usually supported by an annotation tool, such as Brat ^8^ or Knowtator ^9^, which provides a nice graphical user interface and ways to previously configure the annotation schema. A comprehensive survey of the annotation tools for the biomedical domain can be found in Neves *et al.* ^10^. A good approach on corpus construction should include training for the particular annotation tool and the guidelines, an inter-annotator agreement and the construction of a consensus corpus derived from the later.

Frequently, manual annotation is supported by text mining by providing automatically extracted annotations which are later validated by the annotators. This validation process should not only include checking the annotations which were automatically extracted by the text mining tools but also carefully reading the text to identify missing ones. Hybrid corpora in which part of the annotations correspond to non-validated automatic annotations and then manually annotated with others, such as relationships, can also be found. For instance, for the Drug-Drug Interaction corpus ^11^, drugs were automatically annotated using the Metamap tool ^12^ followed by the manual annotation of relationships by experts.

Finally, corpora can also be completely derived from automatized methods and never manually validated by experts, the so-called silver-standard corpora. Despite the undeniable presence of wrong annotations and the absence of many others, previous works have demonstrated that these corpora can support development of semi-supervised or distant supervised systems for named-entity ^13^ and relationship extraction ^14^. As manual annotation or validation is not required in this case, such corpora tends to be much larger than the gold-standard ones. CALBC ^15^ is an example of a silver-standard corpus derived from a community-based project which intended to automatically harmonize annotations generated from a variety of named-entity recognition tools.

In this work, I present a review on 36 corpora which are available for the biomedical natural language processing (BioNLP) domain and perform an analysis on the semantic types which they include. The motivation for this work is to provide the first comprehensive overview on BioNLP corpora and thus support choosing the most appropriate collection whenever necessary. Additionally, I show the impact of each corpora in the field and give insights for the construction of new corpora or for the extension of existing ones.

## Corpora and semantic types

### List of corpora

Here I present a comprehensive study on the semantic entities included in the gold-standard corpora which have been annotated for the named-entity recognition (NER), relationship extraction and event extraction tasks. Although there are corpora available for other BioNLP tasks, such as text classification ^16^ and question answering ^17^, these are not covered in this survey. I focus on gold-standard corpora which contain annotations for entity types, such as genes/proteins, chemicals and species. Thus, I also did not include corpora which have only text span annotations not related to a particular semantic entity, such as the Data Deposition corpus ^18^ which contains annotations on data deposition statements. Given the focus on Biology, I did not consider corpora which were built with the medical domain in mind, such as BioText ^19^ and Variome ^1^. Silver-standard corpora, such as CALBC ^15^, were also not included here. I also do not cover corpora which focused on the linguistic aspects rather on semantic annotations, such as the BioScope corpus ^20^, which contains annotations for negations and speculations statements. Finally, only corpora which are still available for download were included.

In this section, I give an overview of 36 corpora made available on the BioNLP domain and describe how the semantic analysis of the corpora has taken place.

### List of biological corpora

Here I present the list of 36 corpora which have been considered in this study. For each of them, I include a brief description of its origin, which may include the type of documents it contains (abstracts and full texts), its annotation schema, tools which have been based on it, further extensions it has received and the number of citations its publications have received. Table 1 shows a summary of these corpora, including their first publications, year of release (according to the main publication), number of citations according to Google Scholar (as December-2013) and the corresponding URL. Some of the corpora I present here are included in the WBI repository ( http://corpora.informatik.hu-berlin.de), which provides their full visualization using the Stav/Brat annotation tool ^8^. The collections are presented in the alphabetical order.

**Table 1.** Overview of the corpora: main publication, year of publication, citations in Google Scholar (as December-2013) and the URL are shown for each corpus.

Corpus	Ref.	Year	Cit.	URL
AIMed	[ 21]	2005	270	ftp://ftp.cs.utexas.edu/pub/mooney/bio-data/
AnEM	[ 22]	2012	9	http://www.nactem.ac.uk/anatomy/
AZDC	[ 23]	2009	19	http://diego.asu.edu/downloads/AZDC
Bact. Gene Int.	[ 24]	2012	11	https://sites.google.com/site/bionlpst/home/bacteria-gene-interactions
BioCreative GM	[ 4]	2008	126	http://biocreative.sourceforge.net/biocreative_2_gm.html
BioInfer	[ 25]	2007	246	http://mars.cs.utu.fi/BioInfer
CellFinder	[ 2]	2012	5	http://cellfinder.de/about/annotation/
CG	[ 26]	2013	3	http://2013.bionlp-st.org/tasks/cancer-genetics
CHEMDNER	[ 7]	2013	7	http://www.biocreative.org/tasks/biocreative-iv/chemdner/
CRAFT	[ 5]	2012	17	http://bionlp-corpora.sourceforge.net/CRAFT/
Craven	[ 27]	1999	374	http://www.biostat.wisc.edu/~craven/ie/
DDI	[ 28]	2013	0	http://labda.inf.uc3m.es/ddicorpus
EBI Disease	[ 29]	2008	66	ftp://ftp.ebi.ac.uk/pub/software/textmining/corpora/diseases
EDGAR	[ 30]	2000	395	ftp://ftp.ncbi.nlm.nih.gov/pub/tanabe/EDGAR_GS.txt
EPI	[ 31]	2012	14	https://sites.google.com/site/bionlpst/home/epigenetics-and-post-translational-modifications
EU-ADR	[ 32]	2012	4	http://euadr.erasmusmc.nl/sda/euadr_corpus.tgz
GeneReg	[ 33]	2010	11	http://www.julielab.de/Resources/GeneReg.html
Genia	[ 3]	2003	575	http://www.nactem.ac.uk/aNT/genia.html
Genia Ev. Extr.	[ 34]	2008	236	http://bionlp.dbcls.jp/redmine/projects/bionlp-st-ge-2013/wiki/Wiki
GETM	[ 35]	2010	13	http://getm-project.sourceforge.net/
GREC	[ 36]	2009	53	http://www.nactem.ac.uk/GREC/
HPRD50	[ 37]	2007	268	http://www2.bio.ifi.lmu.de/publications/RelEx/
ID	[ 31]	2012	14	https://sites.google.com/site/bionlpst/home/infectious-diseases
IEPA	[ 38]	2002	208	http://orbit.nlm.nih.gov/resource/iepa-corpus
Linnaeus	[ 6]	2010	79	http://linnaeus.sourceforge.net/
LLL	[ 39]	2005	163	http://genome.jouy.inra.fr/texte/LLLchallenge/
Metab. Enzym.	[ 40]	2011	14	http://www.nactem.ac.uk/metabolite-corpus/
MutationFinder	[ 41]	2007	83	http://mutationfinder.sourceforge.net/
Nagel	[ 42]	2009	12	http://sourceforge.net/projects/bionlp-corpora/files/ProteinResidue/
NCBI Disease	[ 43]	2012	10	http://www.ncbi.nlm.nih.gov/CBBresearch/Fellows/Dogan/disease.html
OSIRIS	[ 44]	2008	20	https://sites.google.com/site/laurafurlongweb/databases-and-tools/corpora
PC	[ 45]	2013	4	http://2013.bionlp-st.org/tasks/pathway-curation
PICAD	[ 46]	2011	1	http://stat.fsu.edu/~jinfeng/resources/PICAD.txt
SCAI	[ 47]	2008	57	http://www.scai.fraunhofer.de/en/business-research-areas/bioinformatics/research-development/information-extraction-semantic-text-analysis/named-entity-recognition/chem-corpora.html
SNPCorpus	[ 48]	2011	3	http://www.scai.fraunhofer.de/snp-normalization-corpus.html
Species	[ 49]	2013	1	http://species.jensenlab.org/

***AIMed.*** The AIMed corpus ^21^ contains annotation on proteins and protein-protein interactions (PPI) for 200 abstracts, which were selected from the documents for which curated annotations were found in the Database of Interacting Proteins ( http://dip.doe-mbi.ucla.edu/dip/Main.cgi). The corpus is one of the five corpora widely used for the development of PPI extraction methods ^50^ and thus, has been used for the development of a variety of PPI tools ^51^.

***AnEM.*** The recently published AnEM corpus ^22^ contains a total of 500 documents which contains annotations on the following anatomical entity types: organism subdivision, anatomical system, organ, multi-tissue structure, tissue, cell, developing anatomical structure, cellular component, organism substance, immaterial anatomical entity and pathological formation. It is probably the largest manually annotated corpus on anatomical entities and has been used for the development of the AnatomyTagger tool ^52^.

***AZDC.*** The AZDC corpus ^23^ contains almost 800 abstracts which includes the ones available in the EBI disease corpus (cf. below) and some from the Craven corpus (cf. also below). It contains annotations for diseases and normalization to UMLS unique concepts for some semantic subtypes and was used for the development of named-entity recognition tools for disease names, such as the recent DNorm system ^53^.

***Bacteria Gene Interaction.*** The Bacteria Gene Interaction (BGI) corpus ^24^ was developed in the scope of the BioNLP Event Extraction Shared Tasks 2011 for assessing the extraction of genetic processes in *Bacillus subtilis*. It is derived from the LLL corpus (cf. below) for PPIs. This corpus has been extended for the Gene Regulation Network (GRN) task ^54^ in the 2013 edition of the same challenge.

***BioCreative 2 Gene Mention.*** The BioCreative 2 Gene Mention ^4^ corpus has been used in two editions of the BioCreative challenges ( http://www.biocreative.org/) to foster improvements for gene/protein extraction. It is composed of sentences, opposed to documents, which were derived from Medline documents and contains annotation on gene and protein, though it does not make distinction between them. Given that it has been used in one of the most popular challenges in the BioNLP community, several studies have used this corpus for the development of gene/protein extraction systems, such as BANNER ^55^.

***BioInfer.*** BioInfer ^25^ is also one of the five popular corpora available for PPI ^50^. It contains sentences derived from more than 800 documents and annotations are available for genes, DNA families or groups, proteins, protein complexes and protein families and groups. Just as the other five PPI corpora, the BioInfer corpus has been used for training and evaluation of several tools ^51^.

***CellFinder.*** The CellFinder corpus ^2^ was developed in the scope of the CellFinder database ( http://cellfinder.de/) and includes annotations for six entity types (anatomical parts, cell lines, cell types, species and cell components) for 10 full text documents in the stem cell research field. This corpus has been mainly used for the evaluation of named-entity recognition approaches for the above entity types in Neves *et al.* ^2, 56^.

***Cancer Genetics.*** The Cancer Genetics (CG) corpus ^26^ was constructed for the Cancer Genetics task in the BioNLP Event Extraction Shared Task in 2013 and includes annotations on the development and progress of cancer. The corpus is composed of 600 abstracts split into three datasets and events are composed of anatomical and molecular entities, as well as annotations for organisms.

***CHEMDNER.*** The CHEMDNER corpus ^7^ has been recently created in the scope of the CHEMDNER task in BioCreative IV for assessing performance of named-entity recognition tools for chemical compounds. It contains 10,000 abstracts split into training, development and test datasets and annotations for chemicals are classified in eight categories, such as systematic, formula or abbreviation.

***CRAFT.*** The CRAFT corpus ^5^ is a recent and very comprehensive collection of 97 full text documents which has been annotated with concepts, such as gene/proteins, species, cells and chemicals, from nine ontologies and terminologies. The authors have reserved 30 of the full texts for a text mining challenge that is going to be carried out in the near future.

***Craven.*** The so-called Craven corpus ^27^ is in fact a collection of three corpora which contains annotations on sub-cellular locations, PPIs and gene-disease associations. These corpora have been used for the development of methods for extracting the above binary relationships and support construction of knowledge bases.

***Drug-Drug Interaction.*** The Drug-Drug Interaction (DDI) corpus ^28^ includes more than 700 documents derived from Medline and DrugBank, and includes annotations for drugs and binary relationships between them. It has been already evaluated on two shared tasks ^11, 57^ and thus, has been extensively used for both training and evaluation for NER and relatiosnhip extarction tasks.

***EBI Disease.*** The EBI Disease corpus ^29^ is composed of 600 sentences selected from the Craven corpus (cf. above) which have been extended with associations to unique concepts in the UMLS terminologies.

***EDGAR.*** The EDGAR corpus ^30^ contains annotations for genes, drugs and cells, including binary relationships between genes and drugs, genes and cells, and drugs and cells.

***Epigenetics and Post-translational Modifications.*** The Epigenetics and Post-translational Modifications (EPI) corpus ^31^ was developed for the BioNLP Event Extraction Shared Task 2011 and contains 1,200 abstracts annotated with events related to epigenetic changes. Just like the Genia Event Extraction corpus (cf. below), it contains annotations for genes/proteins and annotations identified as “Entity” which might refer to a variety of entity types, such as cell locations or small molecules.

***EU-ADR.*** The EU-ADR corpus ^32^ was constructed in the scope of the EU-ADR project, which aimed to automatically process health records. The corpus contains a total of 300 abstracts which are split into three groups, each containing annotations for two entity types and binary relationships: drug-target, drug-disease and target-disease.

***GeneReg.*** The GeneReg corpus ^33^ is composed of 314 abstracts related to *Escherichia coli* and contains annotations of events for gene expression regulation. It has been created in order to allow its interoperability with the Genia corpus (cf. below) and other lexical resources, such as WordNet and the Specialist lexicon.

***Genia.*** The Genia corpus ^3^ is probably one of the most popular corpora in the biomedical domain and has been used for the development of many named-entity tools, such as ABNER ^58^, and also to assess systems in a shared task ^59^. It contains 2,000 Medline abstracts with annotations based on the Genia ontology for DNA, RNA, proteins, lipids, cells, tissues, body parts and cell lines, among others.

***Genia Event Extraction corpora.*** The Genia Event Extraction (Genia EE) corpus ^34^ has started from the annotation of 1,000 abstracts, half of the Genia corpus (cf. above), and was annotated with genes/proteins and biological events, such as gene expression and gene regulations. This version of the corpus was used for the BioNLP Event Extraction Shared Task which took place in 2009 ^60^ and then extended with 15 full texts for the following edition of the challenge that took place in 2011 ^61^. A new corpus composed of 34 full texts was constructed for the third edition of the shared task that took place in 2013 ^62^. The corpora have been used for the development and comparison of a variety of systems for extracting events.

***GETM.*** The GETM corpus ^35^ is composed of 150 abstracts derived from the development dataset of the Genia Event Extraction corpus (cf. above). Relationships were annotated between the gene expression events and the annotations for cells and anatomical locations which were present in the original corpus. It was used for the evaluation of a rule-based relationship extraction system on gene expression events in cell locations.

***GREC.*** The GREC corpus ^36^ contains annotations for 240 Medline abstracts for events on gene regulation and expression related to ontologies, such as Gene Ontology and Sequence Ontology.

***HPRD50.*** The HPRD50 corpus ^37^ has been created in the scope of the RelEx system and contains 50 abstracts and annotations for PPIs. The corpus is also one of the five PPI corpora ^50^ and has been used for the development of a variety of PPI tools ^51^.

***ID.*** The ID corpus ^31^ was developed for the BioNLP Event Extraction Shared Task 2011 and contains 30 full text documents annotated with biomolecular mechanisms of infectious diseases. The corpus is split into three datasets (training, development and testing) and events are related to annotations of proteins, chemicals and organisms.

***IEPA.*** The IEPA corpus ^38^ is composed of more than 200 sentences extracted from Medline abstracts and is annotated with binary relationships between proteins. It is also one of the five popular corpora available for PPI ^50^.

***Linnaeus.*** The Linnaeus corpus ^6^ contains 100 full text documents annotated with annotations for organisms, all linked to identifiers in NCBI taxonomy ( http://www.ncbi.nlm.nih.gov/taxonomy). It was built for the development of the Linnaeus system, one of the state-of-art tools for the annotation of organism names.

***LLL.*** The LLL corpus ^39^ for PPI in *Bacillus subtilis* was release in the scope of the Learning Language in Logic (LLL) shared task and was later also included in the package of the five popular corpora available for PPIs ^50^. The proteins are identified as agent or target in the relationships.

***Metabolites and Enzymes.*** The Metabolites and Enzymes corpus ^40^ contains annotations for metabolites and enzymes names in almost 300 abstracts and was used for the evaluation of dictionary-based approaches for the recognition of these entity types.

***MutationFinder.*** The MutationFinder corpus ^41^ is composed of 508 Medline abstracts annotated with mutations and it was used for the evaluation of the homonymous tool based on regular expression techniques.

***Nagel.*** The Nagel corpus ^42^ contains annotations for protein residues, species and mutations in 100 Medline abstracts which have been used for the evaluation of a system developed for the extraction of these triplets.

***NCBI Disease.*** The NCBI Disease corpus ^43^ is composed of almost 800 abstracts derived from the AZDC corpus (cf. above) split into three datasets for training, development and blind testing. Annotations are classified into categories, such as modifier and specific disease, and it has been used for the development of the DNorm tool ^53^.

***OSIRIS.*** The OSIRIS corpus ^44^ contains abstracts annotated with genes and sequence variants and was used for the evaluation of a dictionary-based system developed for the extraction of the later. Annotations for genes are normalized to identifiers from the NCBI EntrezGene database ( http://www.ncbi.nlm.nih.gov/gene).

***Pathway Curation.*** The Pathway Curation (PC) corpus ^45^ was created for the homonymous task in the BioNLP Event Extraction Shared Task 2013 in which participants were required to extract biomolecular events to support curation of pathways. It includes a total of 525 abstracts annotated with events which contain chemicals, gene, proteins, complexes and cellular components as arguments.

***PICAD.*** The PICAD corpus ^46^ is another less popular PPI corpus composed of more that 1,000 sentences which were assembled in the scope of the development of a tool for this purpose.

***SCAI.*** The SCAI corpus ^47^ includes 100 abstracts with annotations for chemicals and training and test datasets for the recognition of IUPAC names. This has been one of the most popular corpora for chemical named-entity recognition and has been used for the development of many tools, such as ChemSpot ^63^.

***SNPCorpus.*** The SNPCorpus ^48^ contains almost 300 abstracts and annotations for protein sequence and nucleotide sequence mutations and it has been used by the authors for extraction of these mentions from the text and their association to identifiers in biological databases.

***Species.*** The Species corpus ^49^ has been recently built as an alternative to Linnaeus (cf. above). Instead of using full text documents, it aimed at providing more variability on the species names by using eight groups of 100 abstracts on the following categories: bacteriology, botany, entomology, medicine, mycology, protistology, virology, and zoology.

## Semantic analysis of corpora

In this section, I show an analysis of the semantic types of the annotations present in the corpora discussed above. This analysis has been carried out based on the publications associated with the corpora and sometimes by checking the annotation types for the corpora which are available at the WBI Corpora repository. Here I only consider those annotations which are meaningful enough to be associated with one of the pre-defined semantic types under consideration (cf. below). For instance, I do not consider the “Entity” annotations in the Genia Event Extraction corpus ^60^.

Six top level semantic types were decided based on the annotations available in the corpora and on the UMLS semantic types ( http://semanticnetwork.nlm.nih.gov/SemGroups/SemGroups.txt). The following are the types along with their mapping to the UMLS sematic groups and types:

- gene/protein: semantic group "Genes & Molecular Sequences" (GENE), as well as the types T116 (Amino Acid, Peptide, or Protein) and T114 (Nucleic Acid, Nucleoside, or Nucleotide);
- variant/mutation: semantic type T045 (Genetic Function);
- drug/chemical: semantic group "Chemicals & Drugs" (CHEM), except for the types T116 and T114 which were considered gene/proteins (cf. above);
- cell/anatomy: sematic group "Anatomy" (ANAT);
- disease: semantic group "Disorders" (DISO);
- organisms: semantic group "Living beings" (LIVB).

The gene/protein category covers a wide range of small molecules and includes gene, proteins, protein complexes, gene complexes, protein families/groups, RNA, DNA families/groups, regulons, etc. Most of the corpora which include these entities do not make a distinction between them, such as the BioCreative Gene Mention ^4^. In the cell/anatomy semantic type, I include all kinds of cellular and anatomical locations, whether *in vivo* or *in vitro*, as follows: cell lines, cell types, cell components, sub-cellular locations, developing anatomical structures, anatomical systems, organs and tissues. Drugs and chemicals were put together in the same group as some corpora include both of them, although these are sometimes classified into categories. Variants and mutations were assembled in a single group and, finally, one category for diseases and one for species, which are more homogeneous groups and whose annotations are not usually classified in distinct categories in corpora.

## Comparison and discussion

**Figure 1.**
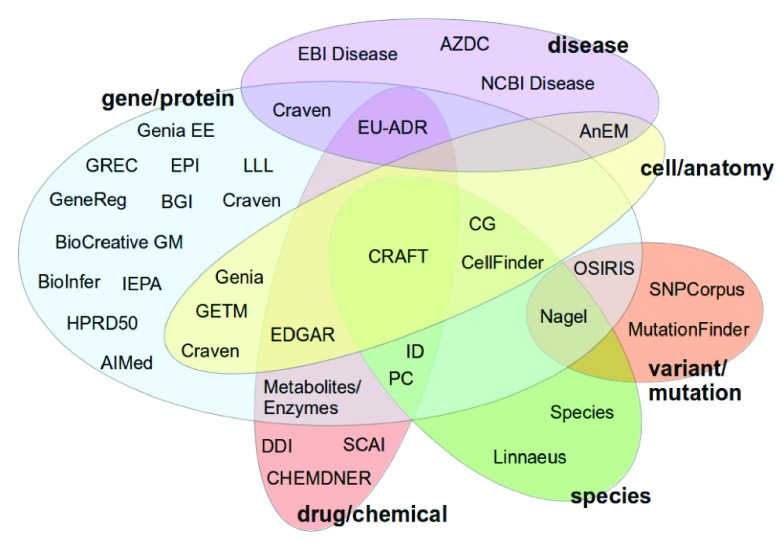
Classification of the corpora according to the semantic annotations they contain.

In this section I present an analysis of the semantic types for the named-entities present in 36 corpora. Figure 1 shows an overview of which annotations are available for each corpora, as well as which corpora contain annotations for a particular semantic type. It also gives an idea of the similarities between corpora in terms of the entity types they share.

The closer a corpus is to the center of the figure, more distinct semantic types it contains. The CRAFT corpus is the collection which contains the higher number of semantic entities, namely, gene/proteins, species, chemicals and cells, but still lacks annotations for disease, variants/mutations and anatomical parts. The Cancer Genetics (CG), CellFinder, EDGAR, EU-ADR, Infectious Disease (ID), Pathway Curation (PC) and Nagel corpora are the ones which come closer to the CRAFT corpus, each containing annotations for three different types, but with a great variability on which of these three types are considered.

On the other hand, the farther a corpus is to the center of the figure, less distinct semantic types it contains and most of the corpora fall into this situation. There are 12 corpora which contain only annotations for gene/proteins, three for diseases, two for variants or mutations, two for species or organisms and three for chemical or drugs. Curiously, no corpus contains annotations only for cell anatomical entities, except the AnEM corpus, which was also placed in the disease semantic types because it contains annotations on pathological formations.

Genes and proteins are the most popular entities in biomedical corpora: in a total of 26 collections. However, these have different purpose and number of documents. Early initiatives, such as the BioCreative Gene Mention corpus, were based on sentences instead of documents, but following corpora have annotated the abstracts instead. Recently developed corpora include annotation of full text documents, such as CellFinder, CRAFT and the Genia Event Extraction, in order to allow systems to make use of the complexities of the languages which can only be found in the full text but not in the abstracts ^64^. Most of the corpora classified in this group make no distinction between genes, proteins, complexes, or families, except for Genia and the Bacteria Gene Interaction corpora. Corpora whose annotations are mapped to identifiers in a database, e.g., EntrezGene, such as CRAFT and OSIRIS, allow their use for the development of gene/protein normalization tools ^65^. Finally, the high number of corpora available for gene/protein corpora is due to the importance of these entities for the molecular biology domain and to the research in the last years on PPIs and biological events.

Corpora which contained annotations for chemicals and drugs were few until the release of the SCAI corpus, which focused initially on the IUPAC nomenclature. But this has become a hot topic in the last couple of years and following corpora have provided annotations also for drugs and their interactions (DDI corpus), as well as anotations on full text documents (CRAFT corpus). The CHEMDNER corpus classifies chemicals in some predefined categories and was used in the one of the shared tasks in last BioCreative challenge, which attracted the participation of many teams. Relationships of chemical compounds with other semantic entities can be found in the EU-ADR and also for more complex events, such as in the shared tasks of Cancer Genetics and the Infectious Disease in the BioNLP Event Extraction Shared Tasks.

During many years, the Linneaus corpus and tool have been the state-of-art resources for benchmarking and extraction of species annotations, respectively. The simplicity of the nomenclature and the high performance of Linnaeus has not encouraged further research in this line. However, the release of the Species corpus some months ago aims to provide more variety on the annotations for organism, by choosing a higher number of abstracts, as opposed to few full text documents in the Linnaeus corpus. Additionally, abstracts are grouped on eight categories of organisms (bacteriology, botany, entomology, medicine, mycology, protistology, virology, and zoology), thus, ensuring the diversity of annotations. Other recent full text corpora which contains annotations for organisms are the CellFinder and CRAFT corpora.

Annotations for cell and anatomical parts have since many years been limited to the cell lines and cell types in the Genia and EDGAR corpora. However, the recent release of the AnEM corpus, which include a careful classification of these entities based on many ontologies, along with the AnatomyTagger tool ^52^, will certainly encourage new solutions in this area. Other recent corpora for cell annotations are the full text documents of the CRAFT corpus, including mapping to the Cell Ontology, as well as the annotations for cell lines, cells types and anatomical parts in the CellFinder corpus.

Most corpora which contain annotations for diseases exclusively are somehow related to each other as all of them contain documents which have been selected from the AZDC and the Craven corpora. The recent release NCBI Disease corpora aims to improve research in this field by classifying mentions based on some pre-defined categories, followed by the release of the DNorm tool ^53^. Associations of diseases with other entity types are still scarce and only present in the EU-ADR and Craven corpora.

Finally, variations and mutations have also received little attention from the BioNLP community, and the four available corpora are composed only of abstracts. Co-occurrence of these entities in the text is available for genes in the OSIRIS corpus, however, no explicit relationships was annotated between them. Such relationships are only available with genes and species in the Nagel corpus, but its small size (100 abstracts) hinders text mining solutions based on machine learning methods, being only suitable for evaluation purposes.

From Figure 1, it is straightforward to observe which corpora are available according to the entity types of interest. The aim of this study is to encourage the use of less popular corpora which are already available and whose suitability for the text mining tasks has been scientifically evaluated. However, when choosing to use more than one corpora, the text miners will probably need to deal with more than one format for the documents and annotations, and write specific parsers for each of them. This is a problem that the BioC initiative ^66^ is aiming to solve with the recent introduction of the BioC XML format. Indeed, many of the corpora shown here have already been converted to this format using the Brat2BioC tool ^67^ and made available in the WBI Corpora repository. Given that most of the corpora are available under a flexible license, this review will also serve as a starting point for further updates on the repository and allow not only their availability for visualization but also for download in the BioC format.

## Conclusions

In this survey I presented an overview on the semantic entity types available for 36 corpora in the biomedical domain. The annotations were classified in six categories (gene/protein, drug/chemical, cell/anatomy, variant/mutation, species and disease) and an overview on which corpora contain each of these semantic types has been shown. I hope that this review can be of help when choosing the best corpora for developing a named entity recognition tool and also to encourage re-use (re-annotation) of existing corpora instead of building a new one.
