# Supplementary material for: An analysis on the entity annotations in biological corpora
Source: F1000Res. 2014 Apr 25;3:96. [Version 1] doi: 10.12688/f1000research.3216.1 (PMC4168744; doi:10.12688/f1000research.3216.1)
Supplement: Peer review history

# Peer review: Referee response for version 1

**DOI:** 10.5256/f1000research.3456.r5114

**Published:** 2014-07-14

**Authors:** Paloma Martínez

**Affiliations:** 1 Department of Computer Science, Charles III University of Madrid, Madrid, Spain

The article shows a review of 36 publicly annotated corpora in the biological domain. The analysis is performed taking into account different features such as type of text (abstracts, full text publications, etc.), types of entities, types of relationships (if any), number of documents, if automatic or manual annotation, if annotations are related to other resources (such as ontolologies), etc.It is an interesting and useful work that can help researchers to find a corpus to perform evaluations of their machine learning methods. As the author describes in the introduction sections, annotated corpora are valuable resources that allow to train and test algorithms and also to compare state-of-the art works each other.I think that the paper is a good contribution to the journal. Below I give several suggestions in order to improve the article as well as some data to correct a couple of mistakes.

- In the Introduction section, it would be beneficial to introduce to the reader the “ *inter-annotator agreement*” as well as the main ways to calculate it.
- Before introducing the annotation schema it is necessary to give a definition of “ *Schema*” (is it a conceptual schema? What are the elements a schema should contain)
- In the paragraph about DDI corpus, some remarks should be included: in the first version of the corpus used in the  DDIExtraction 2011 task the drugs were automatically annotated by Metamap tool ^1^ but in the new version (used in DDI Extraction 2013 shared tasks), every annotation was manually revised by two pharmacists)
- At the end of this section, I would like to see something about the precision experts have annotating entities and relationships. This would also help to know what is the limit systems are able to manage recognizing entities and relations.
- In section “ *Corpora and semantic types*” it is a good idea to give the number of citations from Google Scholar, although the most recent corpora have almost no citations. The DDI corpus has 0 citations because the reference is from October 2013; Segura-Bedmar *et al. *(2011) ^1^is another reference that the author could include in the article corresponding to the first version of the corpus.
- Concerning the figures of DDI corpus, it consists of 792 texts selected from the DrugBank database (DDI-DrugBank dataset) and other 233 Medline abstracts (DDI-MedLine dataset) on the subject of DDIs. The corpus was manually annotated with a total of 18,502 pharmacological substances and 5028 DDIs, including both pharmacokinetic (PK) as well as pharmacodynamic (PD) interactions.
- Concerning drugs, there are two corpora that do not appear in the article: (a) PK Corpus and PF DDI Corpus ^2^ with approx. 600 abstracts about clinical pharmacokinetics and pharmacogenetics, in-vitro and in-vivo drug-drug interactions.  (b) PK DDI corpus ^3^ consisting of 64 FDA drug labels with annotations for drugs with their precipitant or object roles in drug-drug interaction.
- Instead of having a paragraph for each corpus, I suggest including a tabular representation of corpora and characteristics. For instance, with a column for each feature (document type, annotation tool, categories of entities, number of mentions, format, availability, etc.). This representation would help to compare different corpora.
- I also suggest mentioning that linguistic phenomena such as co-reference resolution are also required in annotation task, especially in the detection of entities. For instance, how are words such as “drug”, “disease”, “medication” and others annotated in these corpora?
- Some typos: In the section: ***List of biological corpora - Drug-Drug Interaction:***
* “... *has been extensively used for both training and evaluation for NER and** ******relatiosnhip extarction******** **tasks.*”*

I have read this submission. I believe that I have an appropriate level of expertise to confirm that it is of an acceptable scientific standard.

## References

[1] Segura-BedmarIMartínezPSanchez-CisnerosD: The 1st DDIExtraction-2011 challenge task: Extraction of Drug-Drug Interactions from biomedical texts. *Proceedings of the 1st Challenge Task on Drug-Drug Interaction Extraction.*.2011;761:1-9Reference source

[2] WuHYKarnikSSubhadarshiniAWangZPhilipsSHanXChiangCLiuLBoustaniMRochaLMQuinneySKFlockhartDLiL: An integrated pharmacokinetics ontology and corpus for text mining.*BMC Bioinformatics*.2013;14(35) : 9610.1186/1471-2105-14-3523374886PMC3571923

[3] BoyceRGardnerGHarkemaH: Using natural language processing to identify pharmacokinetic drug-drug interactions described in drug package inserts. *Proceedings of the 2012 Workshop on Biomedical Natural Language Processing*.2012;206-213Reference source

# Peer review: Referee response for version 1

**DOI:** 10.5256/f1000research.3456.r4561

**Published:** 2014-05-06

**Authors:** Roman Klinger

**Affiliations:** 1 Cognitive Interaction Technology – Center of Excellence (CIT-EC), Bielefeld University, Bielefeld, Germany

The paper *“An analysis on the entity annotations in biological corpora”* is a review of several corpora which are available to the research community and which consist of textual data with annotation. These annotations are either on an entity level or with relations between entities.

The author makes very clear what this paper is about, which corpora are discussed and where the border is drawn to resources not discussed here. The comprehensive list of corpora is enriched with citation counts to make clear which corpora are already commonly used and which might be under-explored by the community. She makes clear that she would like to motivate researches to make use of these corpora as well.

In general, I think that this paper is a very valuable review of resources available to the community. The author does not define strictly what the motivation for this paper is, what the audience should be and who can specifically benefit from it. I could think of at least two scenarios:

1. A researcher would like to improve or develop a method for a specific domain/entity class (like drug-drug-interaction or recognition of chemical names, for instance). Then the author can get an overview of available corpora and be quite sure that she or he does not miss a corpus of the specific domain when the class is mentioned in this review.

2. A researcher would like to evaluate a method and needs resources, he or she does not necessarily care so much about the specific domain and can select from the variety of discussed resources in this review.

I propose that such or similar motivations are added to the introduction.

**Title**: I am not sure if a corpus can be “ *biological*” — or dealing with classes from the bio(medical) domain.

**Abstract**: Should indicate for whom this review might be of value. Some examples of cases for use would be great, I think.

**Introduction**: 

- A schema does not consist of entities only, but also of entity classes. 
- Maybe it would be interesting to discuss the issue of having a bias in the annotation towards the automatic tool when annotations are only validated.
- “ *Such corpora tends…”* —> “ *corpora tend*” or “ *such corpus tends*”
- “ *I show the impact of each corpora*” —> “ *each corpus*”
- The author cites the paper on the 5 reviews commonly used to study PPI. Are there no other such reviews?

**Corpora and semantic types:**

**List of corpora:**

- “ *I also did not include corpora which have only text span annotations not related to a particular semantic entity* *…”* — I do not understand that. Several of the corpora the author is discussing do not have links to database or ontology IDs. This should be made clear. (examples are the BC2 GM corpus, the SCAI corpus, I think AZDC as well.)
- Abner is at least evaluated on BioCreative data as well. I am not sure it has only been trained on GENIA.

**Semantic Analysis of Corpora**

- “ *I only consider those annotations which are meaningful enough to be associated with one of the pre-defined semantic types under* *consideration*” I think that formulation could be improved to make clearer what ‘ *meaningful’* means.

**Comparison and Discussion**

- “ *On the other hand*” — without “ *On the one hand*”
- “ *different number of documents*” — “ *numbers*”?
- “ *relationships was*” -> “ *relationships were*”
- “ *corpora which contains*” -> “ *contain*”

I have read this submission. I believe that I have an appropriate level of expertise to confirm that it is of an acceptable scientific standard.
